# Analysis of the impact of three phthalates on the freshwater gastropod *Physella acuta* at the transcriptional level

**DOI:** 10.1038/s41598-021-90934-9

**Published:** 2021-06-01

**Authors:** Marina Prieto-Amador, Patricia Caballero, José-Luis Martínez-Guitarte

**Affiliations:** Grupo de Biología y Toxicología Ambiental, Facultad de Ciencias, UNED, Paseo de la Senda del Rey 9, 28040 Madrid, Spain

**Keywords:** Environmental impact, Freshwater ecology

## Abstract

Plastic pollution is one of the leading environmental problems. Phthalates are widely used plastic additives released into the environment. Although the effects of phthalates on vertebrates have been extensively studied, there is a knowledge gap regarding their effects on invertebrates. This work analyzes the impact of three phthalates, diethyl phthalate (DEP), benzyl butyl phthalate (BBP), and bis-(2-ethylhexyl) phthalate (DEHP), on the gastropod *Physella acuta* at the molecular level to establish the putative pathways involved in its response to them. By real-time PCR, we obtained the expression profile of 30 genes in animals exposed for 1 week to 0.1, 10, and 1000 μg/L of each phthalate. The genes cover DNA repair, detoxification, apoptosis, oxidative and stress responses, immunity, energy reserves, and lipid transport. The results show that while DEP and DEHP did not alter the mRNA levels, BBP modulated almost all the analyzed genes. It can be concluded that the impact of BBP is extensive at the molecular level. However, it cannot be dismissed that the increase in transcriptional activity is a general response due to this compound’s well-known role as an endocrine disruptor. Additional research is needed to elucidate the differences observed in the impact of these compounds on the gastropod *P*. *acuta*.

## Introduction

Plastics are incredibly versatile materials and are useful for a wide range of applications. However, plastic production is under the scope of green policies to reduce the pollution of the environment. It has been estimated that 8300 million metric tons (Mt) of virgin plastics were produced in 2017. In 2015, approximately 6300 Mt of plastic waste were generated, with 79% accumulated in landfills or the natural environment^1^. Plastics production requires specific catalysts and other additives. Phthalates are esters of phthalic acid used as plasticizers to increase the flexibility, transparency, durability, and longevity of plastics, mainly to soften polyvinyl chloride (PVC). As the phthalate plasticizers are not chemically bound to PVC, they can leach, migrate, or evaporate into indoor air and the atmosphere, foodstuffs, and other materials. Their worldwide production increased from 2.7 to nearly 6 million tons per year during the decade of 2007–2017^2^, and they are now ubiquitous environmental contaminants. They are released regularly from the products that contain them^2,3^, and reach almost all the environment's compartments^4^. In German rivers, phthalates have been found from 0.33 to 97.8 μg/L for bis-(2-ethylhexyl) phthalate (DHEP) and from 0.12 to 8.80 μg/L for dibutyl phthalate (DBP), while concentrations in sediment were from 0.21 to 8.44 μg/kg dry weight of DEHP and 0.06 to 2.08 μg/kg dw for DBP^5^. A more recent study found different phthalates in varying ranges in the Ganga River, including dimethyl phthalate (DMP) from 0.03 to 0.05 μg/L; diethyl phthalate (DEP) from 0.04 to 2.14 μg/L; di-*n*–butyl phthalate (DnBP) from not detected (ND) to 2.27 μg/L; benzyl butyl phthalate (BBP) from ND to 0.13 μg/L; bis (2-ethylhexyl) adipate (DEHA) from ND to 0.19 μg/L; bis (2-ethylhexyl) phthalate (DEHP) from 0.11 to 6.3 μg/L; and di-*n*-octyl phthalate (DnOP) from ND to 0.05 μg/L^6^.

The impact of phthalates on the environment has been studied in recent few years, with a focus mainly on vertebrates^7,8^. It is known that phthalates act as endocrine-disrupting chemicals (EDCs), producing severe health effects^7,9–12^ and even have a long-term impact on the epigenome^13^. Phthalates can alter an animal’s metabolism^8^, but there is still a lack of information about their effects on invertebrates. Although the studies often include analysis at the molecular level^14–16^, the diversity of invertebrates demands additional studies involving other species and groups.

Mollusks are one of the invertebrate groups that have been poorly studied, especially freshwater representatives. The few studies have focused on marine species, and the molecular analysis in freshwater species is basically absent. In gastropods, the studies to date have centered on the marine representative *Haliotis diversicolor*^17–19^, showing that phthalates produce oxidative stress and alterations in lipid and energy metabolism. The freshwater snail *Physella acuta* (Draparnaud, 1805), also known as *Physa acuta*, belongs to the Physidae family. It is a cosmopolitan species living in lakes and ponds. It is easily cultured, so it could be a good representative of freshwater gastropods to assess the toxicity of compounds.

In *H. diversicolor*, phthalates can alter physiological processes, such as development and growth^18^. These effects result from the effects at lower complexity levels such as molecular and cellular processes. Therefore, it is essential to obtain information about the mechanisms involved in the effects and the response to phthalates to understand the impact on an animal. The changes in mRNA levels are valuable tools to detect the putative pathways altered by a chemical; this information provides a picture of the damage that it can cause. We recently obtained the transcriptome of *P*. *acuta* and identified genes to analyze the toxicity of the fungicide vinclozolin^20^. Taking advantage of the transcriptome and sequences in the database, we have identified new genes to study several pathways involved in the response to phthalates. The genes are related to DNA repair, apoptosis, detoxification (phase I, phase II, and phase III), oxidative stress, the stress response, immunity, epigenetics, lipid transport, and energy reserve metabolism.

This work aims to offer some insight on the impact, at the molecular level, that phthalates have on the gastropod *P*. *acuta*. Two-month-old adult snails were exposed for 1 week to three phthalates, namely DEP, BBP, and DEHP. The gene expression profile of 30 genes was obtained for the first time in response to these compounds, which is an initial step to assess the damage they can produce in a freshwater invertebrate that is pivotal to the food web of freshwater ecosystems.

## Results

### Gene identification

**Figure 1:**
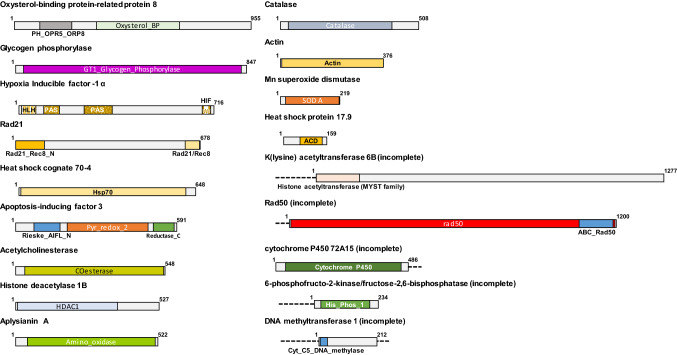
Structure and conserved domains of the identified *Physella acuta* proteins. Each protein corresponds to an open reading frame from the sequences used in the study. The proteins are shown with the different motifs that characterize them. The domains are defined according to the CCD functional classification of proteins. Some of the genes were not complete, and the discontinuous line indicates the unknown up- and down-stream regions.

Eighteen genes were identified for the first time in this work for *P. acuta* (Table 1, Fig. 1). They all showed homology with genes in the database, mainly with those described in the freshwater snail *Biomphalaria glabrata*. In some of the genes, the homology was with *Aplysia californica* and *Aplysia kurodai*, and only one had homology with a gene of the marine bivalve *Mytilus coruscus*. The genes covered several pathways. Two of the genes coded for proteins homologous to RAD21 and RAD50, which are involved in repairing DNA damage^21,22^. Two other oxidative-stress-related genes code for catalase (Cat) and manganese superoxide dismutase (SOD Mn), enzymes involved in removing free radicals^23^. One sequence codes for acetylcholinesterase, an enzyme involved in nerve impulse transmission. The other genes coded for proteins related to apoptosis (apoptosis inducible factor 3 [AIF3]); stress (small heat shock protein 17.9 [sHSP 17.9];, heat shock protein cognate 70-4 [HSC70-4]; hypoxia-inducible factor 1 α [HIF-1α]); histone modification (histone deacetylase 1 [HDAC1]; lysine acetyltransferase 6B [KATB6]); DNA methylation (DNA methyltransferase 1 [DNMT1]); the immune system (L-amino acid oxidase Aplysianin-A [ApA]); one cytochrome P450 (Cyp72a15), energy reserves (glycogen phosphorylase [PYGL]), and lipid transport (oxysterol-binding protein-related protein 8 [ORP8]).

**Table 1:** Accession numbers and homologies of the newly identified genes in *Physella acuta.*

Accession number	Gene	ORF size (aa)	Similarity	Identity-Homology
MW456925	*rad21*	678	PREDICTED: double-strand-break repair protein rad21 homolog—*Biomphalaria glabrata*	85–92%
MW456929	*rad50*	1200	PREDICTED: DNA repair protein RAD50-like—*Biomphalaria glabrata*	68–84%
MW456922	*AChE*	548	PREDICTED: cholinesterase 1-like—*Biomphalaria glabrata*	56–69%
MW456920	*Catalase*	508	PREDICTED: catalase-like—*Biomphalaria glabrata*	82–90%
MW456928	*AIF3*	591	PREDICTED: apoptosis-inducing factor 3-like—*Biomphalaria glabrata*	77–88%
MW456924	*Cyp72a15*	486	cytochrome P450 72A15—*Aplysia californica*	68–82%
MW456930	*DNMT1*	212	DNA methyltransferase 1, partial—*Aplysia californica*	88–91%
MW456923	*KAT6B*	1277	PREDICTED: histone acetyltransferase KAT6B-like—*Biomphalaria glabrata*	49–62%
MW456919	*HDAC1*	527	probable histone deacetylase 1-B—*Aplysia californica*	88–94%
MW456918	*sHSP17.9*	159	PREDICTED: heat shock protein Hsp-12.2-like—*Biomphalaria glabrata*	52–71%
MW456926	*ApA*	522	Aplysianin-A; Precursor—*Aplysia kurodai*	45–65%
MW456921,	*ORP8*	955	PREDICTED: oxysterol-binding protein-related protein 8-like—*Biomphalaria glabrata*	74–83%
MW456927	*PFKFB2*	234	PREDICTED: 6-phosphofructo-2-kinase/fructose-2,6-bisphosphatase-like—*Biomphalaria glabrata*	84–89%
Suplementary material	*SOD Mn*	219	superoxide dismutase—Mn, mitochondrial-like—*Biomphalaria glabrata*	80–88%
	*Hsc70-4*	648	PREDICTED: heat shock 70 kDa protein cognate 4—*Biomphalaria glabrata*	98–98%
	*HIF1α*	716	PREDICTED: hypoxia-inducible factor 1-alpha-like, partial—*Biomphalaria glabrata*	64–73%
	*PYGL*	847	glycogen phosphorylase, brain form—*Aplysia californica*	89–97%
	*ACTB_G1*	376	ACTB_G1—*Mytilus coruscus*	99–100%

aa, amino acids; ORF, open reading frame.

### Expression profile

**Figure 2:**
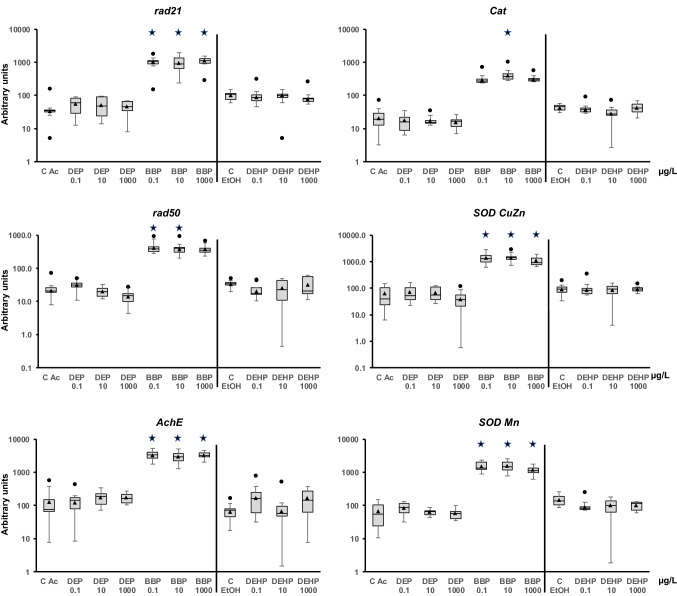
Transcript levels of DNA repairing mechanisms (*rad21* and *rad50*), nervous system (*AChE*), and oxidative stress (*Cat*, *SOD CuZn*, and *SOD Mn*) genes in *Physella acuta* adults after in vivo exposure to diethyl phthalate (DEP), benzyl butyl phthalate (BBP), and bis-(2-ethylhexyl) phthalate (DEHP) for 1 week at 19 °C. The mRNA levels were normalized using *rpL10*, *Act*, *PFKFB2*, and *GAPDH* as reference genes. The comparison was performed with the solvent-exposed (EtOH, acetone) controls. Whisker boxes are shown (n = 9 individuals per box). The median is indicated by the horizontal line within the box, and the 25th and 75th percentiles are indicated by the boundaries of the box. The highest and lowest results are represented by the whiskers. The small triangle inside the box denotes the mean, and the outliers are shown (circles). Significant differences to the respective controls (asterisk) are indicated (*p* < 0.05).

**Figure 3:**
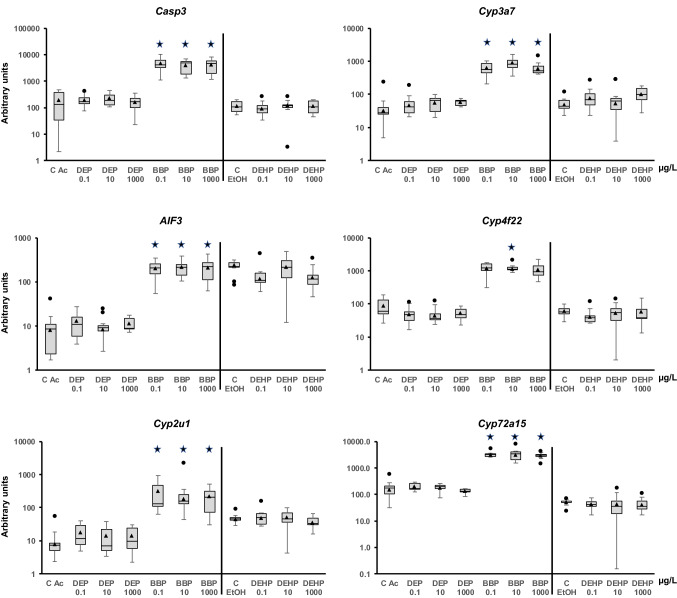
Transcriptional activity of genes related to apoptosis (*Casp3* and *AIF3*) and phase I detoxification (*Cyp2u1*, *Cyp3a7*, *Cyp4f22*, and *Cyp72a15*) in adult *Physella acuta* after in vivo exposure to diethyl phthalate (DEP), benzyl butyl phthalate (BBP), and bis-(2-ethylhexyl) phthalate (DEHP) for 1 week at 19 °C. The mRNA levels were normalized using *rpL10*, *Act*, *PFKFB2*, and *GAPDH* as reference genes. The comparison was performed with the solvent-exposed (EtOH, acetone) controls. Whisker boxes are shown (n = 9 individuals per box). The median is indicated by the horizontal line within the box, and the 25th and 75th percentiles are indicated by the boundaries of the box. The highest and lowest results are represented by the whiskers. The small triangle inside the box denotes the mean, and the outliers are shown (circles). Significant differences to the respective controls (asterisk) are indicated (*p* < 0.05).

**Figure 4:**
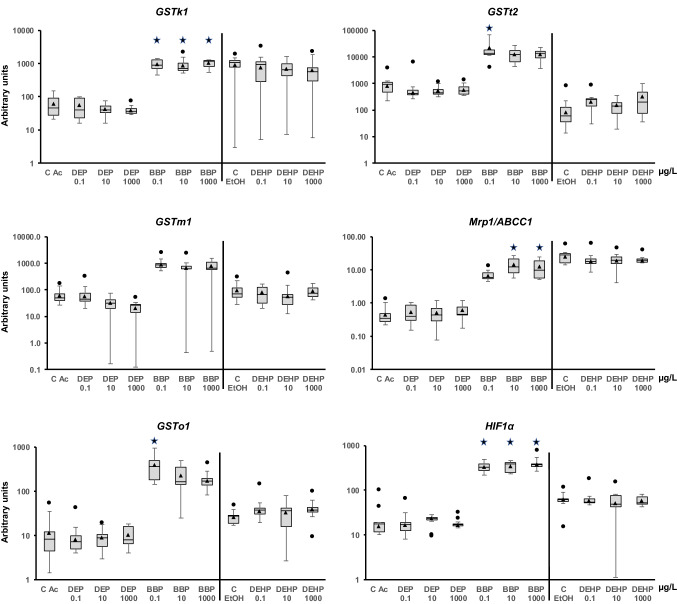
The mRNA levels of genes related to phase II (*GSTk1*, *GSTm1*, *GSTo1*, and *GSTt2*) and phase III (*MRP1*/*ABCC1*) detoxification and hypoxia (*HIF1α*) in adult *Physella acuta* after in vivo exposure to diethyl phthalate (DEP), benzyl butyl phthalate (BBP), and bis-(2-ethylhexyl) phthalate (DEHP) for 1 week at 19 °C. The mRNA levels were normalized using *rpL10*, *Act*, *PFKFB2*, and *GAPDH* were used. The comparison was performed with the solvent-exposed (EtOH, acetone) controls. Whisker boxes are shown (n = 9 individuals per box). The median is indicated by the horizontal line within the box, and the 25th and 75th percentiles are indicated by the boundaries of the box. The highest and lowest results are represented by the whiskers. The small triangle inside the box denotes the mean, and the outliers are shown (circles). Significant differences to the respective controls (asterisk) are indicated (*p* < 0.05).

**Figure 5:**
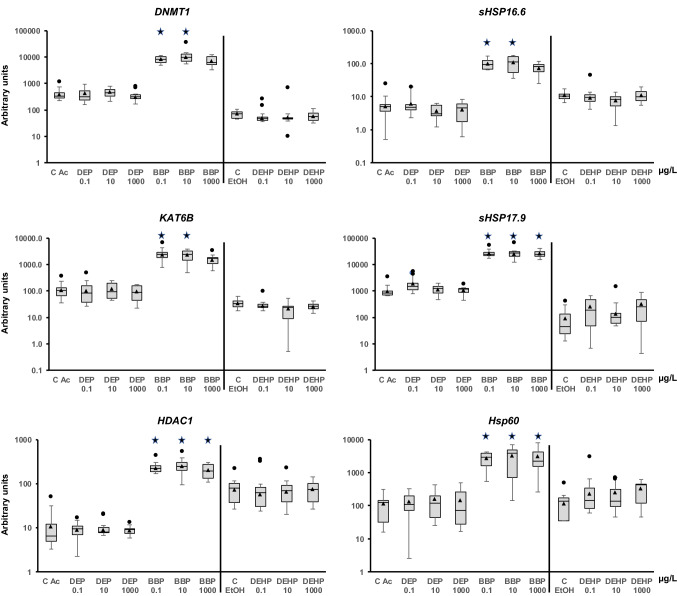
The mRNA levels of genes involved in epigenetic regulation (*DNMT1*, *KAT6B*, and *HDAC1*) and stress response (*sHsp16.6*, *sHsp17.9*, and *Hsp60*) in adult *Physella acuta* after in vivo exposure to diethyl phthalate (DEP), benzyl butyl phthalate (BBP), and bis-(2-ethylhexyl) phthalate (DEHP) for 1 week at 19 °C. The mRNA levels were normalized using *rpL10*, *Act*, *PFKFB2*, and *GAPDH* as reference genes. Treated animals were compared to solvent-exposed (EtOH, acetone) controls. Whisker boxes are shown (n = 9 individuals per box). The horizontal line within the box indicates the median, and the 25th and 75th percentiles are indicated by the boundaries of the box. The whiskers represent the highest and lowest results. The small triangle inside the box denotes the mean, and the outliers are shown (circles). Significant differences to the respective controls (asterisk) are indicated (*p* < 0.05).

**Figure 6:**
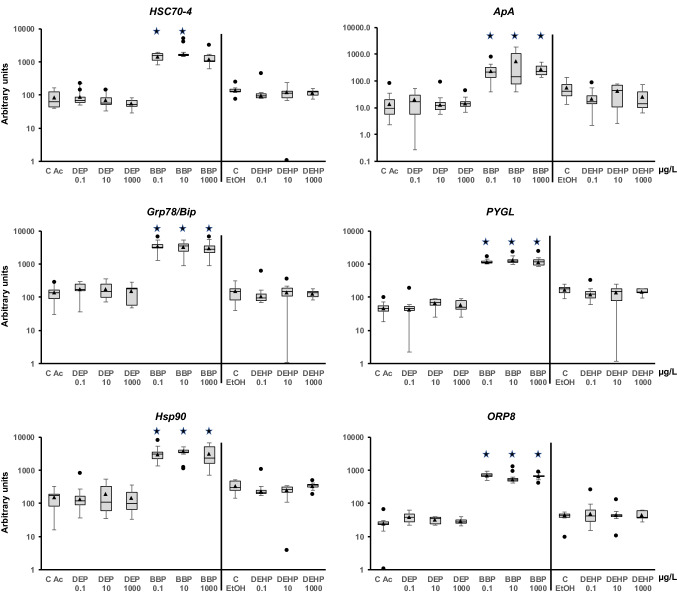
Modulation of genes related to stress (*Hsc70-4*, *Grp78*/*BiP*, and *Hsp90*), immune system (*ApA*), energy metabolism (*PYGL*), and lipid transportation (*ORP8*) in adult *Physella acuta* after in vivo exposure to to diethyl phthalate (DEP), benzyl butyl phthalate (BBP), and bis-(2-ethylhexyl) phthalate (DEHP) for 1 week at 19 °C. The mRNA levels were normalized using *rpL10*, *Act*, *PFKFB2*, and *GAPDH* as reference genes. Treatments were compared to respective solvent-exposed (EtOH, acetone) controls. Whisker boxes are shown (n = 9 individuals per box). The median is indicated by the horizontal line within the box, and the 25th and 75th percentiles are indicated by the boundaries of the box. The highest and lowest results are represented by the whiskers. The small triangle inside the box denotes the mean and the outliers are shown (circles). Significant differences to the respective controls (asterisk) are indicated (*p* < 0.05).

The adult snails were exposed for 1 week to each phthalate. First, the expression of some of the genes changed in response to the presence of ethanol and acetone, as shown in the control samples. The change could be due to greater sensitivity of this animal to one of the solvents. Because the comparison was performed to each solvent control, the putative effect would be neutralized, and the changes observed could be assigned to the presence of the phthalate. The analysis showed that DEP and DEHP did not modify the mRNA quantity of any of the genes at the concentrations tested (Figs. 2, 3, 4, 5, 6). However, BBP was strikingly very effective and altered almost all the analyzed genes, but with some differences. It is worth clarifying that statistical analysis without Bonferroni correction rendered significant differences for all the BBP treatments in all genes except for *GSTm1* (encodes a glutathione S-transferase) at the highest BBP concentration. However, after applying Bonferroni's correction, the transcription of the following genes was increased at all tested concentrations: *rad21*, *AChE*, *SOD CuZn*, *SOD Mn*, *Casp3*, *AIF3*, *Cyp2u1*, *Cyp3a7*, *Cyp72a15*, *GSTk1*, *HDAC1*, *sHSP17.9*, *HSP60*, *Grp78*, *HSP90*, *HIF1a*, *ApA*, *PYGL*, and *ORP8*. Although the remaining genes showed a trend for increased transcription, some results did not show a statistically significant difference from the control. The genes that showed statistical significance for the two lower concentrations but not at the highest were *rad50*, *DNMT1*, *KAT6B*, *sHSP16.6*, and *HSC70-4* (Figs. 2, 5, 6). *Cat* and *Cyp4f22* were altered for the concentration of 1 μg/L but not for the other two (Figs. 2, 3). *MRP1/ABCC1* mRNA levels were increased for 1 and 100 μg/L, while *GSTo1* and *GSTt2* were significantly upregulated for 0.01 μg/L only (Fig. 4). Finally, *GSTm1* did not show statistically significant alterations, although there was a trend for a higher mean compared with the control (Fig. 4).

## Discussion

The development of massive sequencing has provided a relatively inexpensive method to obtain the transcriptome of a species. Taking advantage of this technique, we used a previously obtained transcriptome of *P*. *acuta* to identify 18 genes related to different pathways of interest in ecotoxicology and then examined how exposure to phthalates changed the transcription of these genes. The processes of interest include DNA repair, the stress response, detoxification, apoptosis, immunity, energy reserves, and lipid transportation. There is a growing interest in combining ecologically relevant endpoints with biochemical and molecular parameters to seek a more integrative analysis. In this sense, increasing the number of described genes will allow for the design of standard arrays that could be used in combination with toxicity tests. In this way, initiatives such as the Adverse Outcome Pathway wiki^24^ will increase its relevance in assessing old and new compounds and provide putative mechanisms of action to explain the differences to the animals' specific physiology. Furthermore, increasing knowledge at the molecular level in *P*. *acuta* supports its use as a representative of freshwater gastropods in toxicity analysis. There is a lack of model freshwater mollusks, which is one of the animal groups whose pollution response is currently less known.

The 18 newly identified genes evaluated in this work show homology with those previously described in other species, as expected, mainly with the freshwater snail *Biomphalaria glabrata*, which belongs to the Planorbidae family. *rad21* and *rad50* are both involved in DNA repair: *rad21* is an essential gene encoding a DNA double-strand break repair protein^21^, and *rad50* is a member of the protein complex MRN (including Mre11, RAD50, and Nbs1) that functions in DNA double-strand break repair to recognize and process DNA ends as well as a signal for cell cycle arrest^25^. There is very little information about these genes in mollusks, with only one report in *Crassostrea gigas* for *rad50*^26^. The relevance of these genes is that their detection can be combined with other methodologies, such as the comet assay, to perform an integrated study to determine whether a compound is genotoxic and whether the organism has the ability to compensate for the damage.

The *Cat* and *SOD Mn* genes allow us to evaluate the status of oxidative stress. Oxidative stress analysis is usually focused on biochemical parameters, such as enzyme activity. However, it should also include a transcriptional activity study because it can provide additional information about the mid- and long-term responses. Protein turnover can also be relevant in the response, especially in chronic exposure to toxicants. Detoxification mechanisms are also important to assess the response to toxicants. GST activity is one of the most used methods to assess detoxification^27^, but it does not differentiate between the members involved. The situation is similar regarding cytochrome P450s, which show high diversity with many roles in the cell^28^. Our identification of the *Cyp72a15* gene increases the number of cytochromes 450 s described in *P. acuta*. Evaluating changes in these genes can help to elucidate how the organism can process the toxicants.

The *sHSP17.9* and *HSC70-4* genes extend the battery of genes available to assess the stress response of *P*. *acuta*. *sHSP17.9* is difficult to match with other species’ genes because while they all have an alpha-crystallin domain, there is no other sequence that presently allows for homology to be established. Additional functional studies will help to search for homology. It is worth mentioning that *HIF1α* offers a new aspect of stress related to hypoxia^29^. The stress response mainly focuses on the canonical heat shock proteins, so other mechanisms involved in specific stresses, such as hypoxia, are usually neglected. With the identification of *HIF1a* in *P*. *acuta*, researchers can evaluate the effect of a toxicant on oxygen intake in this species.

The remaining identified genes allow for the analysis of pathways that can also be altered by toxicants, like apoptosis (*AIF3*), the immune system (*ApA*), energy reserves (*PYGL*), and lipid transport (*ORP8*). To our knowledge, in this study these genes have been analyzed for the first time concerning pollution in freshwater mollusks. The last three genes, *DNMT1*, *KATB6*, and *HDAC1*, are involved in epigenetic mechanisms. There is increasing evidence that epigenetic regulation is one of the long-term effects of toxicants. However, the genes involved in this process in invertebrates are still poorly represented in toxicity analysis. The description of these three genes opens the possibility of analyzing their role in the epigenetic response and its relevance in the transgenerational effects that have started to be described with different toxicants^30–32^.

Plastics in the environment are a growing problem. During the degradation process, the polymers themselves and the compounds used as additives, including phthalates, are released. Hence, the presence of phthalates is increasing in the environment^5,33,34^. We analyzed three phthalates in this work, namely BBP, DEP, and DEHP; they showed a differential impact in *P*. *acuta*. DEP and DEHP, did not alter any of the mRNA levels. Researchers have described previously that both phthalates can alter the physiology of invertebrates^16,35–38^, including mollusks^39–41^. Other phthalates can also alter development and growth, which could be related to the endocrine-disrupting activity described for those chemicals. The molecular mechanisms involved are still under investigation, but some data are available. In the clam *Venerupis philippinarum*, DEHP alters the immune response^40^. In *H*. *diversicolor*, DBP affects oxidative stress, lipid and energy metabolism, and osmoregulation^17^. In other invertebrates, including *Chironomus riparius*^42^, *Drosophila melanogaster*^43^, and *Caenorhabditis elegans*^15^, phthalates alter endocrine pathways. The changes affect the ecdysone response as well as the expression of insulin-like peptide. Other pathways are also affected by phthalates, such as oxidative stress and detoxification routes^44^ and the stress response^14^. Finally, in *C*. *elegans,* exposure to environmentally relevant concentrations of diethylhexyl phthalate produces genomic instability by altering the expression of genes involved in DNA repair during meiosis^37^. It is clear then that phthalates can have a broad spectrum of actions in the cell, with a significant alteration of metabolism but primarily affecting oxidative stress and the endocrine system.

The previous studies performed in mollusks have revealed alterations in several physiological processes; the analyzed molecular mechanisms mainly involved oxidative stress and immunity^17,41^. A recent review of the impact of phthalates on aquatic animals summarizes the effects observed, suggesting that activation of the detoxification system (cytochrome P450s) and endocrine system receptors of aquatic animals cause oxidative stress, metabolic disorders, endocrine disorders, and immunosuppression^8^. It would activate a cascade response that could cause genotoxicity and cell apoptosis, resulting in the disruption of growth and development. Considering this, the absence of a response observed in *P. acuta* exposed to DEP and DEHP is striking. The differences observed can be assigned to the type of analysis (molecular vs. physiological), the exposure time (1 week vs. a few hours or days), the concentration used (μg/L vs. mg/L), and evidently, the species used. Additional research will help elucidate the differential response in *P*. *acuta* compared with other organisms. However, it is essential to highlight that the obtained results suggest that *P*. *acuta* can manage the environmentally relevant doses of DEP and DEHP used in this work. This species may be less sensitive to these phthalates, but this eventually will require further research, including the use of other methodological approaches, to confirm it.

In contrast to DEP and DEHP, BBP showed a marked effect: it increased the mRNA levels of almost all the analyzed genes. It is essential to consider that most studies on invertebrates that involve transcriptional activity analysis use arthropods and short exposure times^14,44–46^. Limited data are available on mollusks and, usually, they are marine representatives^40,47^. To our knowledge, this is the first study on a freshwater snail that shows that BBP can produce a substantial effect on cell metabolism. Several of the altered pathways can explain, in some way, the effects observed in other organisms, like DNA repair by the alteration of *rad21* and *rad50*, which are related to DNA damage, or the alteration of the genes involved in histone and DNA modification (*KAT6B*, *HDAC1*, and *DNMT1*), which are related to epigenetic regulation. Apoptosis, which phthalates can also alter, also seems to be modulated in *P*. *acuta* by altering the *AIF3* and the *casp3* genes. Furthermore, the three phases of the detoxification could be acting since the genes tested (three cytochrome P450s, three GSTs, and *MRP-1*) were upregulated.

Genes involved in oxidative stress and the stress response were also altered, as shown by the changes in the mRNA levels of *Cat*, SODs, stress proteins, and the hypoxia-related transcription factor genes. These changes support the alteration of oxidative stress, the stress response, and detoxification, backing previous analysis and adding new insight about the mechanisms involved in modulating these processes. In this sense, the absence of changes in *GSTm1* supports a differential role for each GST family member in the response to toxicants. The altered acetylcholinesterase mRNA level also suggests effects in the nervous system, requiring additional research to elucidate the damage to the central nervous system. Finally, the alteration of *PYGL*, *ApA*, and *ORP8*, involved in energy metabolism, immunity, and lipid transport, respectively, shows that *P. acuta* responds to BBP in a way that has been observed in other organisms. In summary, the present gene profile obtained in response to BBP in *P. acuta* supports the proposed mechanisms and cellular processes in studies with other animals^8^. Immunity, oxidative stress, the stress response, detoxification, apoptosis, epigenetic modulation, DNA repair, lipid metabolism, and energy metabolism are modulated. The nervous system could also be affected. Of note, some genes showed differences in transcription based on the phthalate concentration. These findings suggest there are subtle differences, and additional kinetic analysis is required to elucidate early and late activated genes and the relevance of the damage for the population's future.

The obtained results are in line with previous studies in other organisms, which have confirmed that BBP can induce different types of damage such as apoptosis^48^, genotoxicity^49^, oxidative stress^50^, stress response activation^45^, or endocrine disruption^14^. Although there are studies in invertebrates showing the impact on development and other physiological processes^39,51^, most of them did not focus on the putative mode of action, with only a few of them trying to delve into the response mechanisms. Here we have shown that BBP can extensively affect the cell transcriptional activity in *P. acuta*. These results could be considered to reflect specific alterations on these pathways. This scenario would mean that BBP is the most active phthalate in *P. acuta*, with a broad spectrum of action and a potential effect on many pathways. However, the more probable picture is something that has been recently proposed: alterations in the oxidative stress response and the endocrine system cause a cascade of responses that affect different pathways and ultimately block growth and development^8^. It is relevant to keep in mind that BBP is a known endocrine disruptor^47^. A recent study in *Daphnia magna* provides some insight. Specifically, RNA-Seq revealed that genes involved in signal transduction, cell communication, and embryonic development were significantly down-regulated, while those related to biosynthesis, metabolism, cell homeostasis, and redox homeostasis were remarkably upregulated upon BBP exposure^46^. Although the organism and the stage analyzed are different from our study, those results support the idea that BBP can simultaneously alter multiple pathways, and it fits better with the regulatory role of the endocrine system and the extensive affection by oxidative stress.

As stated before, the results obtained in this work show that DEP and DEHP had no apparent effect to *P*. *acuta* after 1 week exposure to environmentally relevant concentrations. However, BBP showed a strong effect. The difference in response could be due to several reasons that need to be explored in future work. One possibility is the structure of each compound. In this sense, BBP has two benzene rings while DEP and DEHP have only one. This factor could determine the biological activity of these compounds. Another possibility is that DEP and DEHP have effects earlier than the time studied, and the cell returned to the basal state, being able to process and remove the compounds. Finally, it cannot be dismissed that DEP and DEHP are not toxic to *P. acuta*, at least at environmentally relevant concentrations. In any case, BBP alters the metabolism of this species and produces a broad impact on different pathways. Additional research should be done in *P. acuta* and other freshwater species to determine the impact on organisms based on the freshwater ecosystem food web.

## Conclusions

The phthalates DEP and DEHP had no apparent effect on 30 genes in adult *P*. *acuta* (the mRNA levels were similar in the treated and control animals). However, BBP strongly affected almost all the genes and thus appears to have an extensive action that alters DNA repair, apoptosis, epigenetic regulation, the stress response, immunity, and energy metabolism. BBP is toxic for *P. acuta* at the environmentally relevant concentrations used. However, additional research is needed to elucidate the kinetics and the extent of the response. Additional research is also required at different time points with DEP and DEHP to confirm that they cannot induce responses at the concentrations used. Finally, 18 of the analyzed genes have been described for the first time in *P. acuta*. They increase the number of pathways that can be analyzed and support the use of this species in assessing toxicants in freshwater mollusks.

## Materials and methods

### Chemicals and reagents

Diethyl phthalate 99.5% (DEP), benzyl butyl phthalate 98% (BBP), and bis-(2-ethylhexyl) phthalate 98% (DEHP) were acquired from Sigma (Spain). Stock solutions for each compound were prepared at 10.78 mg/mL for BBP, 11.44 mg/mL for DEP, and 965.3 mg/ml for DEHP. The stock solutions were prepared in acetone for BBP and DEP, while DEHP was diluted in ethanol. Exposure solutions were prepared by 1:10,000 dilution of these stocks in artificial pond water (see the *Treatment* section below).

TRIzol and M-MLV enzyme were obtained from Invitrogen (Germany), oligonucleotide dT18 primer and gene-specific primers were supplied by Macrogen (Korea), RNase-free DNase was purchased from Sigma, DNA polymerase and dNTPs were obtained from Biotools (Spain), and EvaGreen was purchased from Biotium (USA).

### Animals

The exposed animals were adults of the freshwater snail *Physella acuta*. The origin and the maintenance of the animals have been described previously^52^. The exposures were carried out in glass vessels filled with artificial pond water prepared with distilled water (2 mM CaCl_2_, 0.5 mM MgSO_4_, 0.77 mM NaHCO_3_, and 0.08 mM KCl). The animals were fed twice a week, once with a 1:1 mixture of Sera Micron and Sera Shrimp Natural (Sera) and once with Sera Shrimp Natural.

### Treatment

Each compound was tested at three concentrations, and six animals (0.091 ± 0.01 g and 0.79 ± 0.08 cm) were exposed for each concentration and experiment. A glass vessel with 300 mL artificial pond water was used for each concentration and compound; in the control, the same amount of solvent (30 μL) was added. The artificial pond water was replaced after three days, and the animals were fed with 20 mg Sera Shrimp Natural (Tetramin, Germany) per recipient. The concentrations used were 0.1078 μg/L (0.345 nM), 10.78 μg/L (34.5 nM), and 1078.0 μg/L (3.45 μM) for BBP; 0.1114 μg/L (0.501 nM), 11.14 μg/L (50.1 nM), and 1114.0 μg/L (5.01 μM) for DEP; and 0.0965 μg/L (0.247 nM), 9.65 μg/L (24.7 nM), and 965.3 μg/L (2.47 μM) for DEHP. Three experiments were performed for each compound, and the animals were exposed for 7 days. At the end of the experiment, three of the animals were frozen in separate tubes for RNA extraction. The concentrations have been named as 0.1, 10, and 1000 μg/L to simplify the labeling in the figures.

### Gene identification

Thirty-four genes were analyzed. Sixteen have been previously described (Table 1)^20,52^. From the other eighteen, two are reference genes (actin beta/gamma 1 and 6-phosphofructo-2-kinase/fructose-2,6-bisphosphatase) while the other 16 belong to DNA repair mechanisms (double-strand-break repair protein RAD21, *rad21*; DNA repair protein RAD50, *rad50*), the nervous system (acetylcholinesterase, *AChE*), oxidative stress (catalase, *Cat*; Mn superoxide dismutase, *SOD Mn*), apoptosis (apoptosis-inducing factor 3, *AIF3*), detoxification mechanisms (cytochrome P450 72A15, *Cyp72a15*), epigenetics (DNA methyltransferase 1, *DNMT1*; lysine acetyltransferase 6B, *KAT6B*; histone deacetylase 1B, *HDAC1*), stress response (sHeat shock protein 17.9, *sHSP17.9*; heat shock cognate protein 70 4, *Hsc70-4;* hypoxia-inducible factor-1 alpha, *HIF1α*), the immune system (aplysianin-A, *ApA*), energy metabolism (glycogen phosphorylase, *PYGL*), and lipid transport (oxysterol-binding protein-related protein 8, *ORP8*).

The sequences were obtained following the procedure described in Aquilino et al.^20^, from the same transcriptome and the sequences obtained by Romiguier et al.^53^. The transcriptome sequences were deposited in GenBank with the accession numbers indicated in Table 2, while those from Romiguier et al.^53^ are included in Supplementary Material.

**Table 2:** Primer sequence and efficiency of the primer set for each gene.

Primer	Primer sequence	Efficiency (%)	Primer	Primer sequence	Efficiency (%)	References
rad21 F	CCGGCCAATGTCTGATGACT	96.9	rpL10 F	TGCACGTGAGGCTGATGAAA	102.3	Aquilino et al.^20^
rad21 R	GCAATTGCTTGCTGGCATCT		rpL10 R	GTGGCCACTTTGTGAAACCC		
rad50 F	AGGCAAGGAGGAGCTACAAC	98.7	GAPDH F	ATACATCAGGAACAGGGACTC	93.9	
rad50 R	TTCAGCCAATGCTAAGCGGA		GAPDH R	GACTTATGACAACCGTGCA		
AChE F	AGTGTCCCGTCGTGGATTTC	89.8	Casp3 F	GTCTGTGTAATTCTCACCCATG	107.2	
AChE R	CACGACCTCGATCTCGTAGC		Casp3 R	AGTTCAGTGCCTCTGCAAGC		
Catalase F	CCCAGTCAGTGGTGATGTCC	98.8	Hsp16.6 F	GCATGAGGAGAAGCAAGACA	96.4	
Catalase R	TTCAGGTGCCCGACAATGTT		Hsp16.6 R	CAGTACACCATGGGCATTCA		
SOD Mn F	TCGAATTGCTACCTGTGCCA	105.4	MRP1 F	CAGGGGCAGGTAAGTCATCC	94.5	
SOD Mn R	ATTCACGTAGTCAGCTCGCA		MRP1 R	AGTGAGCCTTGATCGCACAT		
AIF3 F	ACCACAAGATGCCAACGCTA	102.0	Hsp90 F	GTTTGTGTCACTAAAGAAGGCC	91.8	
AIF3 R	ACTGGCAGCCTTATCAGCAA		Hsp90 R	TGTCACTAGCCTATTTGATACAACC		
Cyp72a15 F	AGGGAAGTGGCTTGAGTGAC	91.9	cyp2U1 F	GTGCATCCTCTACGCGATCA	102.1	
Cyp72a15 R	GGTGCTCAGCCAGCATAAGA		cyp2U1 R	GGCTAGTTTGGGCCTGTCTT		
DNMT1 F	GACGCCATGTCCGATTTACCT	93.3	cyp3a7 F	ACGGCTTGGCCTCTCAATAC	84.8	
DNMT1 R	TCATCCGCGCTGCCACCAG		cyp3a7 R	CGGTTTCTTTCTCGGCGTTC		
KAT6B F	CTTCCATGGGGATGACGAGG	95.1	cyp4f22 F	AGCAGAAAAAGCTCAGCCCT	87.2	
KAT6B R	AAGCTTTGAACGTTTGCCCC		cyp4f22 R	CTTGGTTTTGGCAGCCAGTC		
HDAC1 F	CCCATCAAACATGGCCAACC	90.8	GSTo1 F	CCACCTGGCAACTTGGTTTG	92.8	
HDAC1 R	GTGCATGTGGCAACATTCTGA		GSTo1 R	GCTTGCCAGATGCGTAAGAC		
sHSP17.9 F	TTCACGCGTTGGTGAATCAG	102.5	GSTt2 F	TCGATCTTCTATCGCAGCCG	86.0	
sHSP17.9 R	TTAGCAGCTACAGTCAGCGT		GSTt2 R	TTCTGAGCGCAACAGGTTTG		
Hsc70-4 F	TGGTGTGCCCCAGATTGAAG	107.8	SOD CuZn F	AGAAAGCTGGTGCTGCAACTA	104.9	
Hsc70-4 R	TCCTCTTTGGACAGACGACC		SOD CuZn R	AGGATTAAAGTGGCCTCCAGC		
HIF1a F	AGGATAGATGCTGGCACACC	81.4	GSTk1 F	TGAGCAGAGTAGTTTGGCTGC	96.7	Alonso-Trujillo et al.^52^
HIF1a R	CATAGACACGGTCCTCCCCT		GSTk1 R	ATGCCCCTAATTCTGTGGCT		
ApA F	GTGCCGGGAAAGTGATTTTAG	99.6	GSTm1 F	ATTGGGCCATTAGAGGGCTT	93.1	
ApA R	CAGCCACCAGGGTCGCGA		GSTm1 R	GTTGGACCATCTCCTTGCAC		
PYGL F	ACTGACCCCTGCGTGTAAAG	104.2	Hsp60 F	ATTGCTTATCGTGGCTGAGG	82.0	
PYGL R	TGGAGGCAGGGTTGATCTTG		Hsp60 R	TGGCAATAGCCATATCCTGC		
ORP8 F	GCTGGACGGACATCACTTGT	98.9	Grp78 F	TGGTGGCTCAACCCGTATTC	96.8	
ORP8 R	TGGATGTCTACCACTCGGGA		Grp78 R	CCCCACTCAAAACACCAGCT		
ACT F	GAAGAGCTACGAGCTTCCCG	102.1				
ACT R	CATGGATACCGGCAGACTCC					
PFKFB2 F	AGCGCACTATCCAAACTGCT	110.6				
PFKFB2 R	TCTGCAAAGTCCTGGGGGTA					

### RNA extraction

The frozen snails were used to extract the RNA using TRIzol (Invitrogen, Germany) following the manufacturer's instructions. The animals, including the shell, were homogenized in 300 μL TRIzol. Once the RNA was isolated, it was treated with RNAse-free DNAse for 45 min at 37 ºC to remove the rest of the DNA. Afterward, a phenol: chloroform extraction was performed with Phase Lock tubes (5 prime, USA) to remove any DNAse. The RNA was precipitated and resuspended in 100 μL of diethylpyrocarbonate (DEPC) treated water. After checking the integrity by agarose gel electrophoresis and the quantity by a spectrophotometer (Biophotometer, Eppendorf), the samples were stored at -80 ºC until the next step.

### Reverse transcription

Reverse transcription was performed with the M-MLV enzyme (Invitrogen, Germany) following the manufacturer's instructions. For each sample, 5 μg RNA was used for a 40 μL reaction. The reaction was carried out with poly-dT(18) and 200 units of M-MLV. The mixture was incubated for 50 min at 37 ºC and stopped at 65 ºC for 15 min. The samples were stored at – 80 ºC until use.

### Real-time PCR

The primers were added to the wells, and a master mix of cDNA (0.2 µL/well), 0.5X EvaGreen, 20 units of Taq polymerase, 0.4 mM dNTPs, and 2 mM Cl_2_Mg was prepared. The final reaction volume was 10 µL and was performed with a CFX96 thermocycler (Bio-Rad, USA). The thermal cycling program included an initial denaturation at 95 °C for 30 secons followed by 40 cycles of 95 °C denaturation for 15 s, 58 °C annealing for 15 s, and 72 °C elongation for 30 s. Finally, a melting curve was generated to confirm the presence of a single peak. The reference genes were glyceraldehyde-3-phosphate dehydrogenase (*GAPDH*), ribosomal protein L10 (*rpL10*), actin (*act*), and 6-phosphofructo-2-kinase (*PFKFB2*). Because some of the genes were in low quantities, efficiency curves could not be prepared with the cDNA. Hence, an alternative approach was used. A PCR with the same conditions as RT-PCR for each gene was carried out, and electrophoresis was run to ensure that single products were obtained. One microliter of each gene PCR was mixed in a tube, and water was added to 50 µL (1:50 dilution). From this 1:50 dilution was taken 1 µL and diluted in 500 µl (1:25,000 final dilution). Then, 1 µL was used to prepare the first concentration of the five 1:2-dilution series prepared for the efficiency curve. Primers and efficiencies are listed in Table 2. The RT-PCR was done running duplicate wells for each sample, and two independent replicates were used for each experiment. Bio-Rad CFX Maestro software was used to analyze and determine total mRNA levels of normalized gene expression (2^−ΔΔCq^).

### Statistical analysis

Statistical analysis was performed using SPSS 25 (IBM, USA). Normal distribution and variance homogeneity were tested by the Shapiro–Wilk and Levene tests, respectively. Because the data were not normally distributed, they were analyzed with the nonparametric Kruskal–Wallis test with the Bonferroni correction. Statistical significance was set at p ≤ 0.05.

Figures were prepared by using Excel 365 and Powerpoint 365 (Microsoft, USA).

## Supplementary Information

Supplementary Information.
