# Supplementary material for: Analysis of the impact of three phthalates on the freshwater gastropod Physella acuta at the transcriptional level
Source: Sci Rep. 2021 Jun 1;11:11411. doi: 10.1038/s41598-021-90934-9 (PMC8169832; doi:10.1038/s41598-021-90934-9)
Supplement: Supplementary file 1 — Supplementary Information. [file 41598_2021_90934_MOESM1_ESM.docx]

**Analysis of the impact of three phthalates on the freshwater gastropod *Physella acut******a* at the transcriptional level**

Marina Prieto-Amador, Patricia Caballero and José-Luis Martínez-Guitarte

Grupo de Biología y Toxicología Ambiental, Facultad de Ciencias, UNED, Paseo de la Senda del Rey 9, 28040, Madrid, Spain.

**Sequences were obtained from:**

**Romiguier, J., Gayral, P., Ballenghien, M. et al. Comparative population genomics in animals uncovers the determinants of genetic diversity. *Nature* 515, 261–263 (2014). https://doi.org/10.1038/nature13685**

**● Contig2675 - actin beta/gamma 1**

AAAAAGTACTCTGCGTTGATACCACTGCTTAAGCAGTGGTATCAACGCAGAGTACGCGGGGACCAGACTCTTCGTAAGCCAGCGAAGGTGCCCAGACCGACGTACTTCTTGTAACTCCCCAACAACAACCACACATCACC**ATGTGCGACGATGAAGTAGCTGCCCTCGTAGTGGACAATGGCTCCGGCATGTGCAAAGCCGGGTTCGCCGGAGACGATGCCCCCAGGGCAGTCTTCCCATCCATTGTCGGCAGACCCCGTCATCAGGGTGTCATGGTTGGTATGGGACAGAAAGACAGCTACGTAGGAGACGAGGCCCAGTCCAAGAGAGGTATCCTGACCCTCAAGTACCCCATCGAACACGGCATCGTCACAAACTGGGACGACATGGAGAAAATCTGGCACCACACCTTCTACAATGAGTTGAGAGTCGCCCCCGAGGAGCACCCCGTCCTCCTCACTGAGGCCCCCCTC~~A~~AACCCCAAAGCCAACAGAGAAAAGATGACCCAGATCATGTTCGAGACCTTCAACGCCCCCGCCATGTACGTCGCCATCCAGGCCGTACTCTCCCTGTACGCCTCTGGCCGTACCACCGGTATCGTGCTCGACTCTGGTGATGGTGTCACCCACACTGTCCCCATCTACGAAGGTTACGCCCTCCCCCACGCCATCCTCCGTCTGGACTTGGCCGGCCGTGATCTCACCGACTACCTGATGAAGATCCTCACCGAGAGAGGCTACTCCTTCACCACCACCGCTGAGCGTGAGATTGTCCGCGACATCAAGGAGAAACTCTGCTACGTCGCCCTGGACTTCGAGCAGGAAATGGCCACCGCCGCCTCCTCCTCCTCCCTCGAGAAGAGCTACGAGCTTCCCGACGGTCAGGTCATCACCATTGGCAACGAGCGCTTCAGGTGCCCAGAGTCCCTCTTCCAACCATCCTTCTTGGGTATGGAGTCTGCCGGTATCCATGAAACCACCTACAACTCCATCATGAAGTGCGACGTCGACATCCGTAAGGATCTGTACGCCAACACCGTCTTGTCTGGAGGCACCACCATGTTCCCAGGTATTGCTGACCGTATGCAGAAGGAAATCACCTCCCTGGCCCCCAGCACCATGAAGATCAAGATCATTGCCCCACCAGAGCGTAAATATTCCGTATGGATCGGTGGCTCCATCTTGGCCTCCCTCTCCACCTTCCAACAGATGTGGATCTCCAAACAGGAATACGATGAGTCTGGCCCCTCCATCGTCCACCGCAAGTGCTTC**TAAACGCACTCGCTTTCAAAAAAACGCAACACCACCATCGAAACCATCAAAAAACTTCTTTTATTTTCTACCTATATCAGAACAGTTTCACGATCTCTCGGTGGCTTTTAGGACTTTGATGCCACTGGGAGCCCACTAAAACACGTCGACAACTAACACCAGGGTCGTGGACTGAGAAAGAAGTTTAAGCTATCATTAAAAACTTGTAAACATATTGGTGTGTATCTTAGAACTAATTGCGTCACACTTTGTAAACAAAATTTGTAAGAATGTTTGTGTAGAACTTAAATTGAGTCCAAATTGTACTTTCTAAGTACCGAACTCAAGCATTTCTTTCCAATTTTTCCCCCAAAAACTCATGCCTTCTTTAATTTTAAAATCTTTTCGTTTAGAAATTTCGAGATTTAATGTCGACATTTTAAAAAAGTTTCTGAACGAGAGCACCAACAGGCATTTCAATTTTTGAACAAACCGAAGGCTACAGAAATTAAACCTCAGGAAGTTGTCTGAAAATCTTTAGTCATCCAAATCTTTAGGCTTGCCCACGAAGGGCGAGTAATTTAATCAACTCAAATATTTCAGATTTTTTTTCACGATTTCCTTTTGTAATTTTGTTTCAAACGTAACGAATTCTTATGATCTATGCGCGTACGATTGCGGTCAAGTATGTCATTTTGTGTATGGTGAACGGTCAGCGTTAGTATGTTGCCAGTCGAAGTGTCTCAAAGTTACTCGTAACATCATATCGCGTCAACAAAAGTCGAATAGAAAGTCTGGTCCCGCAGTAACATCATTCATAACTCGTTTACAAACTCTCCACCCGTAAACACAATTGGTCCCTTCGCTTTGATACACTCGCTGGAATCAGACACGATGACGTCTTGCTTATGCAAATGACGATGACGCGCGCCATTACTAATGGAACCCTCCCGCCAGGGCGTCGCCATGGCAACGCCTCGCGTTTCCATAGCGACAGCCAATCTGAAACTCTCGATGACTGATTGGTTACTGTCCCCAGCAAGCTACTGTAGACCTTCGTAAAGGCAGAATAAATCGTGTTGAACAGAAGAGTGCTTGTTTTGTTGCCATTTTTTGCTTGAGAATAAAATGTGTACATCT

**The A marked in blue was deleted to get the ORF which correspond to actin.**

**Protein sequence**

MCDDEVAALVVDNGSGMCKAGFAGDDAPRAVFPSIVGRPRHQGVMVGMGQKDSYVGDEAQSKRGILTLKYPIEHGIVTNWDDMEKIWHHTFYNELRVAPEEHPVLLTEAPLNPKANREKMTQIMFETFNAPAMYVAIQAVLSLYASGRTTGIVLDSGDGVTHTVPIYEGYALPHAILRLDLAGRDLTDYLMKILTERGYSFTTTAEREIVRDIKEKLCYVALDFEQEMATAASSSSLEKSYELPDGQVITIGNERFRCPESLFQPSFLGMESAGIHETTYNSIMKCDVDIRKDLYANTVLSGGTTMFPGIADRMQKEITSLAPSTMKIKIIAPPERKYSVWIGGSILASLSTFQQMWISKQEYDESGPSIVHRKCF

**● Contig3326 - Heat shock protein cognate 70 (4)**

TACACATCTGTACTAGAAAAATCTTCGGGATCCGCCCTCTCTTTCTTGGTTATTTAAGAGCCTTCCATACTCCTCTCTAGAAGTAAATCTGAATTACTGTGCGAGTCTAGCCGGGTGATTTTTTCTGGTTGATGCTGATATAGACCTCTATTACACATACAAA**ATGAGTAAAGCACCTGCTGTTGGTATTGATTTGGGAACCACATACTCCTGTGTGGGTGTATTTCAACATGGCAAAGTTGAAATCATTGCTAATGACCAGGGCAACAGAACAACTCCAAGTTATGTTGCTTTTACAGACAATGAACGTCTTATAGGTGATGCTGCAAAGAACCAGGTAGCCATGAACCCAGAAAATACTGTATTCGATGCCAAAAGATTGATAGGACGTCGTTTTGATGACCCAACTGTAGCTTCCGACATGAAACATTGGCCATTTACAGTATTAAATGAAGCAGGCAAGCCTAAGATCAGGGTTGAATACAAGGGTGAAAACAAAACTTTCTTCCCTGAAGAAATATCCTCAATGGTTCTTACAAAGATGAAGGAAACTGCTGAGGCATATTTAGGAAAGACTGTCACAGATGCTGTTGTTACTGTACCAGCCTATTTCAATGACTCTCAACGTCAAGCTACCAAAGATGCTGGAACTATTTCTGGTCTCAATGTACTTAGAATAATCAATGAGCCAACAGCTGCTGCCATTGCCTATGGTCTTGACAAGAAGGTTGGCGGTGAACGCAATGTACTCATTTTTGACCTTGGAGGTGGCACCTTTGATGTTTCTATTCTTACTATTGAGGATGGCATTTTTGAGGTCAAGTCCACAGCTGGTGACACTCATTTAGGTGGTGAAGATTTTGATAACAGAATGGTCAATCACTTCATTCAGGAATTTAAGAGAAAACACAAGAAGGATATTAGTGAAAACAAGCGTGCAGTACGTCGTCTAAGAACAGCTTGTGAGAGGGCTAAGAGAACTTTATCATCATCTACTCAGGCTAACATTGAAATAGACTCTCTGTTTGAGGGTATTGATTTCTACACCAGTATCACCCGTGCTCGCTTTGAGGAGTTGAATGCTGATCTATTCCGTGGCACCTTGGAACCTGTAGAAAAATCATTGCGCGATGCTAAACTTGATAAAGCTCAGATTCATGAAATTGTGCTTGTTGGAGGATCAACTCGTATTCCAAAGATCCAGAAACTATTGCAAGATTTCTTCAATGGCAAGGAACTGAACAAGAGCATTAATCCAGATGAAGCAGTTGCATATGGCGCAGCTGTACAGGCTGCTATTCTACATGGTGACAAATCTGAAGAGGTGCAGGATCTTCTGTTGTTGGATGTTGCTCCTTTGTCACTTGGTATTGAAACTGCAGGAGGTGTGATGACTGCACTTATCAAGAGAAACACCACTATTCCTACAAAACAGACTCAGACATTCACTACTTATTCAGATAACCAACCTGGTGTGCTAATTCAGGTTTATGAAGGTGAACGTGCAATGACAAAGGATAACAACTTGCTCGGCAAATTTGAACTGACAGGTATTCCACCAGCACCACGTGGTGTGCCCCAGATTGAAGTCACTTTTGACATTGATGCCAACGGTATTCTAAATGTATCTGCTGCTGACAAGAGCACTGGCAAAGAAAACAAAATTACCATCACCAATGACAAAGGTCGTCTGTCCAAAGAGGAAATTGAAAGAATGGTTAACGATGCTGAGAAATACAAGAATGAGGATGAAAAACAGAAAACTCGCATTTCAGCCAAGAATGCTTTGGAAAGTTACTCATTCCATATGAAGTCAACTGTTGAGGATGAAAAACTGAAGGACAAGATCAGTGCAGATGACAAGAAAATTATTATTGACAAGTGCAATGAGATCATTCACTGGCTTGATGCAAATCAGTTGGCTGATCAAGAGGAATTTGAGCATAAACAAAAAGAAATTGAAGGAGTTTGTAATCCAATCATCACCAAATTGTATCAAGGAATGGGAGGTGCTGGTGGCATGCCAGACTTCACTAGTGCTGCAGGTGCTGGTCATGGAGCATCCCAAGGTACAGGAGGCAGTGGACCAACCATTGAAGAAGTTGATT**AATCTGTATAAAGACCTAGTCTAATTTATAGAACTTAGTTAAAAAACCTTTTTGGCTATTTTGCTACGCTGAGCACTAATATCATCCAAAATTTTTACAACTATTTATTTCTATCAACACAATTTAGCTGTTATGTTGCAAATTTG

**Protein sequence**

MSKAPAVGIDLGTTYSCVGVFQHGKVEIIANDQGNRTTPSYVAFTDNERLIGDAAKNQVAMNPENTVFDAKRLIGRRFDDPTVASDMKHWPFTVLNEAGKPKIRVEYKGENKTFFPEEISSMVLTKMKETAEAYLGKTVTDAVVTVPAYFNDSQRQATKDAGTISGLNVLRIINEPTAAAIAYGLDKKVGGERNVLIFDLGGGTFDVSILTIEDGIFEVKSTAGDTHLGGEDFDNRMVNHFIQEFKRKHKKDISENKRAVRRLRTACERAKRTLSSSTQANIEIDSLFEGIDFYTSITRARFEELNADLFRGTLEPVEKSLRDAKLDKAQIHEIVLVGGSTRIPKIQKLLQDFFNGKELNKSINPDEAVAYGAAVQAAILHGDKSEEVQDLLLLDVAPLSLGIETAGGVMTALIKRNTTIPTKQTQTFTTYSDNQPGVLIQVYEGERAMTKDNNLLGKFELTGIPPAPRGVPQIEVTFDIDANGILNVSAADKSTGKENKITITNDKGRLSKEEIERMVNDAEKYKNEDEKQKTRISAKNALESYSFHMKSTVEDEKLKDKISADDKKIIIDKCNEIIHWLDANQLADQEEFEHKQKEIEGVCNPIITKLYQGMGGAGGMPDFTSAAGAGHGASQGTGGSGPTIEEVD

**● Contig5639 - Mn superoxide dismutase**

TACTCTGCGTTGATACCACTGCTTAAGCAGTGGTATCAACGCAGAGTACGCGGGGAATTCAAGGACGGACAAAAACAACA**ATGTTGTCAACAGCTTCACACGTATTAAAAAGATGCCTACAGACATCAGCCACTCGACTGAAACACTCACTGCCAGACTTAAAGTATGATTTCAATGCACTGGAGCCCTACATTTCAGCTGATATTATGAAACTTCATTATGAAAAACATCATCAGGCCTATGTTACCAACCTGAATGTTGCTGAAGAGAAGCTCAATGAAGCTGTTGCCAAAGGAGATCTGAATACTGTCATTAGTCTTCAGCCAGCTCTCAGGTTCAATGGTGGAGGTCACATCAACCACACAATCTTCTGGTCCAACCTGAGCCCCAAGGGAGGCGGCGAGCCCTCAGGCGACCTCATGCAGCAGATTAAAGACGATTTCACCACTTTTGAGCGGATGAAGAAAGAACTTGTGGCCGCCTCTGTAGGTATCCAAGGCTCGGGCTGGGGATGGCTGGGCTACAGTCCAACAAATGGACATCTTCGAATTGCTACCTGTGCCAATCAAGATCCCCTTTTTTGCTACAACAGGTTTGATTCCTATTTTTGGCATTGATGTGTGGGAACATGCTTATTACCTGCAGTATAAAAATGTGCGAGCTGACTACGTGAATGCAATCTTCAATGTGGCCAACTGGGCCGATGTTTCTAATAGATTGGCCAAGGCCAGGATGAAC**TAGAGTCCTGAGATAATATCTCTGAATGCTCACTTTTCTAAAAGAGTAGACTAAAAGTTTGAAAGAATTTTGACAGTCCTTGTCAAATAAGGGGTTCTCACTTTGTGCATCATGCAATGAGTTGTTTCAGTGGGGATAAGAATACTGCCTGCTGGTGATAACTTTATATTACTAGTTATAATTTAAACTAGTTATGTAAAGTTATTATCATGATTTTACATTGTAAAGTGCTCCACATGATGGGTGTTATGATATTAACGTTTATGCAATAAGTTAGTAAACAGAAAGGTGTTTTGTTTACAATTTAAGTAGATGAAAGGTGTAGGGATGTTATGTGAATTTTTCTTTGAGGGCAGTGATACTATTGTGTCATTGTTTAGGAATGATTTAGTACACTGTGTCAGTTGTCTGACCACTAAGTCTCTGCATCTTATTCATTATTTTTTTATTGCTTGACAGATGACTTTATTAAAGTTTAACTGTTGGATAAACTTGTAATTTTATTTCTTTTTCTTTTTGTAGAGGACTAAAACTAATTACTACTGCTAAAGTATTTGTAAATTTACTTTTTGTAGTTTTGATTGCAAGAAGGAGGACTTCTTAACATTTTTCTTTTGTGGTAATGTTATGGATTTTCTTCTGTTAAAAGTGTAACCTTAAATAAAAAGAACCTACAAATAAACGAAAAAA

**The T marked in blue was deleted to get the ORF which correspond to Mn SOD.**

**Protein sequence**

MLSTASHVLKRCLQTSATRLKHSLPDLKYDFNALEPYISADIMKLHYEKHHQAYVTNLNVAEEKLNEAVAKGDLNTVISLQPALRFNGGGHINHTIFWSNLSPKGGGEPSGDLMQQIKDDFTTFERMKKELVAASVGIQGSGWGWLGYSPTNGHLRIATCANQDPLFATTGLIPIFGIDVWEHAYYLQYKNVRADYVNAIFNVANWADVSNRLAKARMN

**● Contig23606 - Glycogen phosphorylase**

GGAGTTCACATTCGGTTGAGGCCCTTATAATAAGATCTGTGGCCTTGCAATTTCAAGCAAATTAGGTGAATATAACAGTCTAGTCGATAGGAAGCATCGACTGAAATCCATTTGTACAACTTATATTTCGAGCATTTAGGGAAAAGCAAG**ATGACATCTAAACAACATGATTCAGATAGACGGAAGCAGATAAGTATAAGAGGGATTGCACCGGTGGAAAACGTAGCAACCATAAAAAAATCGTTCAATCGGCATCTGCATTATACACTTGTTAAAGACAGAAATGTTGCAACACCGAGAGATTATTATTTCTCTCTTGCATACACTGTTCGAGATCATCTTGTATCTCGATGGATTAGGACACAACAGTATTACTATGAAATCGATCCAAAGAGAGTCTATTACCTCTCTTTGGAGTTTTACATGGGCCGCACTCTATCCAACACAATGGTCAACCTGGGCATTCAAAGCTCCTGTGATGAAGCCATGTACCAGCTTGGCTTGGATATAGAAGAATTGGAAGAAATTGAACAAGATGCTGGTCTTGGAAATGGTGGTTTGGGACGTCTGGCTGCCTGCTTCCTTGACTCCATGGCAACTCTTGGCCTTGCTGCATATGGTTACGGCATTCGTTATGATTATGGTATCTTTGCCCAGAAGATTGAGAATGGTTGGCAGATTGAGGAGCCTGATGACTGGCTGAGGTTTGGCAACCCTTGGGAGAAGTCTAGACCAGAGTACTGCCTTCCCGTCAACTTCTTTGGTCGTGTTGAGAACATCAATGGCAAGGATAAGTGGGTGGATACTCAGGTGCTTTTTGCAATGCCTTTTGATACACCCATCCCAGGCTATGGAAACAACACAGTGAACACACTTCGCCTTTGGTCTTGCAAGGCACCAAACAGTTTCCACCTACACTTCTTCAACAACGGTGAATACATCAATGCTGTGTGTGAGAGGAACTATGCTGAAAACATTTCCAGAGTGCTTTACCCTAATGACAACATGTTTGAGGGAAAAAAGTGCCGACTAAAACAAGAATATTTCTTAGTGGCAGCGTCCCTGCAAGATCTGATCCGTCGTTTTAAGTCGGCCACGTTTGGCCAGTCCAACCCAGTGAGAACATCATTCGACACTTTCCCTGATAAAGTAGCCATTCAGCTGAATGACACTCATCCATCACTTGCCATCCCTGAGCTGATGCGCATTCTGATTGATATCGAGAATCTGCCCTGGGAGAAGGCATGGGAACTTACAAACAAGACGTGCGCGTATACCAACCATACAGTTCTTCCCGAAGCTTTGGAACGTTGGCCCGTATCACTATTAGGACATGTTCTACCTAGACATCTAGCTATTCTATTTGAAATAAATTCACGCTTTTTAGCGGAGGTTGCCAAGAAGTGGCCAGGAGACAATGATCGTCTAGCTCGTATGTCTCTGGTAGAGGAGGGACCTGAGAAGAAAATTAATATGGCAAATGTGTGTATTGTAGGAAGCAAGGCAGTGAATGGGGTTGCTGCCATCCATTCTGATATTCTCAAGAAAACAACTTTCAAAGATTTCTATGAGATGTTCCCAAAGAAGTTTCAAAACAAAACCAATGGCATCACCCCAAGACGATGGCTCTTATTGTGTAACCCAGGATTGGCTGATGTCATTGCTGAGAAAATTGGCGAGAGCTGGGTGACCAATTTGACTGAGCTGCGAAAACTGACCCCTGCGTGTAAAGAAGAGGCCTTCTTGCGCAGCTTCATGAAAGTTAAACAAGAGAACAAAATGAGATTAGCCCAGTTTATTCAGACAAATTACCACATCAAGATCAACCCTGCCTCCATGTACGACATGCAAGTGAAGAGGATCCACGAGTACAAGAGACAGTTGCTCAACTGCCTGCACATGATTGTACTCTACAACAGACTCAAGAAGAACCCTCAAGCTCCTTTTGTACCCAGAACTATTATGATTGGTGGAAAGGCAGCACCTGGGTACCATTTAGCCAAGATGATCATCAAGCTGATCAACAACGTTGGCAGAGTTGTCAACAACGACCCCATCATCGGAGATCGCCTGAAAATTGTGTACCTTGAGAACTACCGTGTGTCAATGGCTGAGAGAATTATTCCAGCCGCCGACCTGAGTCAGCAGATTTCTCTAGCTGGCACTGAGGCTTCTGGTACAGGAAACATGAAGTTCATGTTGAACGGTGCTCTCACCATTGGAACACTTGATGGTGCCAATGTGGAAATGATGGAAGAAATGGGCAGAGAAAATATCTTCATCTTTGGTATGACTGTGGAACAGGTGGAGGAGCTATGGAGAAAGGGATACAACCCAAGATCCTATTACGAGAAGGATGCTGAGCTGAAACAAGCTCTGGACCAATTAAACAGTGGCTACTTCTCCCCAGAGGAACCTGGTCTCTTCTGTGAACTCTTCAATGGCTTGCTGAACAATGACAGATTTGCATTGTGTGCAGACTTTAGGGCCTATGTTGATTGTCAACAGGAAGTCTCTGAACTATTCAGGGACCCCATGAAATGGGCTGTGAAGGCAAGCTTGAACGTTGCCTCTGCTGGTAAATTCTCCAGTGACCGAACCATCAATGAATACGCCAAGGATATCTGGGGCGTTGAACCTTCAAACATCAAGCTGCCCAACCCGGCTGACAAAGGCTCTGCTAAGGAGGAT**TAAATCATCTTAGTTGTTTAGTTTTCTACACTAATGAATTATGTAGCAGTTTCTTTCTAGCTAAATTATTTTCTTAATGGAGCTTGGCTGGACTAGAGGGCTTAGGATTGTCTATGATGTGATATACACTTTGTGATTTGTCTTTTAAAACAATCAAAGTATGTGATGCCATATTATTTAAAGTAAATGATCATTATTTCTTTTTTACAAATTTATTCTATATGACAAACCTCAATTCCCCAGAATTGTTTTTGGTTTTTCTGCCAAAAAAGTTTTTGTCCGTATATTGAGTGAATACTTACTTATGGAAGGTTTAGAGCAAATTACTTGAAGTTTAGCTTTATGTTTTGATGTCCATGCATTTAATGCTTCATGTCTTAAAGCTTAAAGTTTTCCGGAATACCTTCGTGACTTTCCCAGATGTGAATGTTTTAGAGTCACTTTAACTGATATACAAAACTTTGTGACTATTTTTAGTTTTTCTCAGGTCCCATCACAAGATAATGGAAAAAAAAAAAATTCTACAGACTTGAATGTCTGTAACTTGAAAAATAACAGCAAACCGCAGTCGTAAAAAATAAAGTTACAAATGTGTTCACCTAACATCTCTGCTGTCTGAAAAAAGTTACAATAAAAACATTTTACTTTTTAAACCATGTCTATATTTTTTTGTCTCCCTTGGTTTTTTAAAAACCTGTACATAGTAGATTTAAGAACTTAAATCAATGTTTTATTTTATTTTTTTTCAACTTTAAAGTTTATATTATCCTTTATTAAAATGTATTTAATAAAGTATTTTCCCCATCTTTCCTATTGAAGTCCTGTGGATTTAGATCCATGTAATCAGACTAGTAGAGAACTAACTGTATCAATTACTAGTTTTACATACTCCTGCACTTAATTATTTTAGTTATCTACCCTGTATGTCTATGTGTGTGAATCTCGTTTTCATATTGTCAAAAGTTTTTTTCACAGGAAGAAACAGTCAAATTTTAACTATGTTCTAATTATTTTCTTTTGTTTTTGCTTTGGATGCTGTACATTATTTTATAATTTTACACCTTTTATGTTGGTTTTTTTTTTGGCTCAGTTTTACAAACCGTTTCTACATATTGTAGACTTTGAAAACCAAATAACAAATATATGAAATCTTAAACCCATGAACCATTGAACATGTCAGAAAGAATAGAATTTATACTAGAAGTTTTTTCTTGTTGTTGTACATCTGTTTATTCTGTGCTTACCATATCTAGTAAAATTTTATACAAACTTTGAACTAGTAGATGCTTTGATTAAATCATTGATGATTGCACAAATATTATATTTTTGGTGAGTAATGATGGAGTTAAGTCCCCTGATTGTTGATGACTTCATTCCCCTGATTTAAACTTAATGGTAAAATTGTTGAAAAATGTATTATACACATGGAGTTCAGTCCCTTGGTTGTTAACTCCAGTTCCCTGGCTTGTTGCTTAATTAAACCAAAGAATTAGTGTTTAAAAAATTGGTTTTTTGAAAAGTTGATTCTCCAGATGGAGTTTAGTCCCTTTATTTTTGATGCCTTAAGTCCCCTGATTTTTTAATTGTTGAAAAGTTGATGCTACATAAGTTCAGCCCTTTGGTTGTTAGTTGAGTATAAAGCTGTGCCTGGTTGATAAAGACTTTGTTTACCAGTGTGCAGTTGAAGGTGTTTGTGATGTGACCATAGATAGATAGACTTGGTAGTATTCTTGAGTGATAAACTTATATTGAAAAGTTGTCAAACTTAGTAGTATTCTTCAGTGGTAACTTTGTATTGAGAAGTTTTCAACCTGAATGGATGTCTTGGCTAGAGCCACACTCACTAGCAGCTTAAGTTGGTCAATCAGTGGTCATTGTGTTGAGTACTCTGGGTTAGCTACTTATGTTTTTTAGCTACTTATGTTTTTCACTGAAAGTGTGGGTCCCTTTAACTGGGTCCCACTGACAGTGTAGGTCCCACTGAATGTGGGTCCCACTGAAAGTGTAGGTCCCTTTAACTGGGTCCCACTGACAGTGTAGGTCCAATAACATTGTAGGTCCCACTGAAAGTGTGGGTCCCTTTAACTGGGTCCCACTGAAAGTGTGGGTCCCACTGAAGGTGTGGGTCCCTTTAACTGGGTCCCACTGACAGTGTAGGTCCCACTTAAAGTGTGGGTCCCTTTAACTGGGTCCCACTGAAAGTGTGGGTCCCACTAAAAGTGTGGGTCCCACTTACTCGGTCCCACTTCCCACTAACTGGGTCCCACTGAGTGGTTCCTTTGCTGGGTGAGTGGGGTGTATGGTACGGGAATCATCTGATTTAATCCTCTAGAAACCCAGGTTTAGTCCTTGACCTTAGTTGATCCACTACAGGATACTTCAACTAGGCAGTGTTTTCTCCCCTCATGTTTTCAGTTAGATTTTTAAATATCTATTGAATAAAGAGGAACATAACAGG

**Protein sequence**

MTSKQHDSDRRKQISIRGIAPVENVATIKKSFNRHLHYTLVKDRNVATPRDYYFSLAYTVRDHLVSRWIRTQQYYYEIDPKRVYYLSLEFYMGRTLSNTMVNLGIQSSCDEAMYQLGLDIEELEEIEQDAGLGNGGLGRLAACFLDSMATLGLAAYGYGIRYDYGIFAQKIENGWQIEEPDDWLRFGNPWEKSRPEYCLPVNFFGRVENINGKDKWVDTQVLFAMPFDTPIPGYGNNTVNTLRLWSCKAPNSFHLHFFNNGEYINAVCERNYAENISRVLYPNDNMFEGKKCRLKQEYFLVAASLQDLIRRFKSATFGQSNPVRTSFDTFPDKVAIQLNDTHPSLAIPELMRILIDIENLPWEKAWELTNKTCAYTNHTVLPEALERWPVSLLGHVLPRHLAILFEINSRFLAEVAKKWPGDNDRLARMSLVEEGPEKKINMANVCIVGSKAVNGVAAIHSDILKKTTFKDFYEMFPKKFQNKTNGITPRRWLLLCNPGLADVIAEKIGESWVTNLTELRKLTPACKEEAFLRSFMKVKQENKMRLAQFIQTNYHIKINPASMYDMQVKRIHEYKRQLLNCLHMIVLYNRLKKNPQAPFVPRTIMIGGKAAPGYHLAKMIIKLINNVGRVVNNDPIIGDRLKIVYLENYRVSMAERIIPAADLSQQISLAGTEASGTGNMKFMLNGALTIGTLDGANVEMMEEMGRENIFIFGMTVEQVEELWRKGYNPRSYYEKDAELKQALDQLNSGYFSPEEPGLFCELFNGLLNNDRFALCADFRAYVDCQQEVSELFRDPMKWAVKASLNVASAGKFSSDRTINEYAKDIWGVEPSNIKLPNPADKGSAKED

**● Contig117514 - hypoxia-inducible factor 1α**

TTTTTTTATGAGGTTACCGGAAACGGAATCAGGGTTCTTAGGAAGACTGTGACGTCAAAGTATCACTACTAAACAAAGTGTGTTTGAAGGATATAATTTTACATAATTTTGAATCAATAAACAAGCTAAACATTCGTGGATGGTTTTTTAAGT**ATGGCCAAAGACAAAAGAAAGAACACAGAGAAGCGCAAAGAAAAGTCTCGAGATGCCGCACGCTGTAGACGAGGGAAGGAGACGGAGGTCTTCATGGAACTCTCCAACTGTCTGCCCATGGCCGAGAGCATATCCAGTCAGCTGGACAAGGCATCCGTCATGAGACTGTCCATCAGCATGCTAAAGATTTACAATATTCTCAACAACACTTTCTACAAATCTGAAGATGGCAAGAGTTTGTCCAAGAAGTCCAAGGGAGATAAGCTGGACCAGCTGTATCAGAAAGCCCTGGAAGGCTTTGCCTTTATCCTGTCCCAGGAGGGAGATATTGTCTACCTGTCTGAGAGTGTCACAAAGTACCTGGGTCTTCAGCAGATTGAGCTGATGGGTCAGAGTATCTACGAGTTCACCCACCCATGTGACCATGATGAAATCAAAGAAATGTTAACAGTGAAACCAAGCGCACAACAAAAGACCCCCAGCAACACAGAGAACAGAATCTTCTTCCTCCGCATGAAGTGCACCCTCACAGCTAAAGGCAGAAATGTCAACCTTAAAAGTGCTACTTTTAAGGTGATGAAATGTTCCGGCCGCCTGGTGACCAAAGAACTTTCCAGTGAAACTGACGTCAGTGCCAGCACCTTCCCCTACCTGGTGGCAGTAGCAGAGCCCATCCCTCACCCTGCCAACATTGAGATCCCCCTGGACAGCAAGACCTTCCTGTCCAAACACAGCATGGACATGCACTTTATCTTCTGTGATGAAAGAATTGAAGAGCTAGCCGGCTACAATTGTGAAGCTATGATTGGCCAGTCCCTGTATGACTTCCACCATGCTCTGGACTCAGATGTCATTGACAAAGCCTTTAAAGACCTTTTCTCTAAAGGCCAGACAATGACTGGAGCCTACCGTTTCCTGGCCAGGCATGGTGGGTACATGTGGGTCATCACACAAGCAACTATCATCAACAACAGCCGTACACAAAAGCCACAGTGGGTTGTGTGTGTACACTATGTTCTGAGCCAAGTGGAGGAGAGAGGGAGCATCCTGTCCCATGTACAGACAGACGAGGCCCCCCTGTCTGACCTGATGCCACCCAAGCTGGAGCTGAGTACTGAGAACATCTTCGCCCCCAAGACCAAGGACATGGACACTGGTTATTTTGTACCACCTGAGCTCAAGAATTCTTTAACTTTCCTAAAGAAAGATGAACCAGAAGATTTGTCCTACCTAGCACCAAATGCAGGGGATGAAAGTGTGCCTCTATATAACTATGGAACAGATGCACTGCTGCTGTGTGCCAGCCCCCCACTGAAGACAGAACCAGAGAACAGCTTGCTGGACATGTGTTACCGCAAGGACAACTCCCCCCTCTCAGCCCTCTCTTCTAATGCCTCCTCCAGGATCGCCAGCCCAAGTGACTACCTGAACATTGCAGTACCAGGAGATGTGGAGTCCATGGACCAGTTCTTCCAGTCCATCAAGGCCTCGGACCAGGATCCTGATGTGGAGGACATTGATTTCGACATGAGAGCGCCTTATATACCCATGGATGGGGACGAGGACTTGGGGCTACTGCCTCCGTCTTCAAACATCCTCTTCAATCTTTCATCAGATCTAAACCCAGGATTATTTGGACAAACAGAATCAGTATTTGGACCCAAACATGCCCTGTTTGAGGAGCTGCCCCAACCCCCTAAAGCCAGTGTCCGGGACATGCTAGGGGGCAGCACTGCTGTGGCCAGTATAGAGCAGCCCCCTGACACCATGTACCTCCAGATGAAGAGACCTCTGGACATGAATAGCCTTGAGAACGGTCCACCAACCAGGAAGGTCATTAGGATAGATGCTGGCACACCTGATATACAAACTAGTGCCAGTACTCTACAGACAGACAGTGGAGGTGGTGGCTGTAACAAAGACAGTGTACTCCTCAACCTCCTCCTGAGAGGGGAGGACCGTGTCTATGGTTACAAGGTCAACAACATGATTGGCAAGACATTCAGTCAAGCAATGCTACCAAACTTGACCCGACAAGACTGTGAAGTCAATGCTCCTATTCAAGCTAATCCCCTGCTTCAAGGGAGGGAACTGCTTCATGCACTAGAGGGGATCAAAATGGGAGCAAAGATTGTCCGCCCCCACCTG**TGAGGTGATACCCTGGAATGTGCTGTTGATGTTCCATGAAACAGATATTTGTCCATTTGATGACATCAAAAGAATGAGGCTATTTTTTTCACCCACCATTTTCTGCTGGATGCTGGTCCCAGTTTTTTTACAGCAAGCAGCTTTTTAAGGAATTAAAGACACAACTGACCAAACAGGAATTCAAGTGCTGGGATTCAAGTTTAATTTGCCGTTACAAAGTGGACCATATTAGGAGACAAACATTTGAAGGCAACTTTGGTCATGGCTTAGTGCTGAGACCTAAGTTATAAAACTATAAGATAGGCATGTACATATAGCATGGGTGGATTCTTCTCTTTATTCAACTAGACTGGAAACTAATATCTCAATGGCCTTATTCCCACTTGGTGAAATTCAATTATTTTTTACTCTTTTAGTGTACAAAAAAAAATCTCCCTTTCATTACATTGTTTTTATTGTGCTGACAAAAATGTTAAGAAGTAAAAAAAATTTGTAGAATAATTTTTTTTTTTAAAATTTATTAGTTGCGCCAACTGGGAGACCAAGATGAGTGCATTTATATCTTTCAATGTTGAAGGCATGTTCCTTATAGTCTACAG

**Protein sequence**

MAKDKRKNTEKRKEKSRDAARCRRGKETEVFMELSNCLPMAESISSQLDKASVMRLSISMLKIYNILNNTFYKSEDGKSLSKKSKGDKLDQLYQKALEGFAFILSQEGDIVYLSESVTKYLGLQQIELMGQSIYEFTHPCDHDEIKEMLTVKPSAQQKTPSNTENRIFFLRMKCTLTAKGRNVNLKSATFKVMKCSGRLVTKELSSETDVSASTFPYLVAVAEPIPHPANIEIPLDSKTFLSKHSMDMHFIFCDERIEELAGYNCEAMIGQSLYDFHHALDSDVIDKAFKDLFSKGQTMTGAYRFLARHGGYMWVITQATIINNSRTQKPQWVVCVHYVLSQVEERGSILSHVQTDEAPLSDLMPPKLELSTENIFAPKTKDMDTGYFVPPELKNSLTFLKKDEPEDLSYLAPNAGDESVPLYNYGTDALLLCASPPLKTEPENSLLDMCYRKDNSPLSALSSNASSRIASPSDYLNIAVPGDVESMDQFFQSIKASDQDPDVEDIDFDMRAPYIPMDGDEDLGLLPPSSNILFNLSSDLNPGLFGQTESVFGPKHALFEELPQPPKASVRDMLGGSTAVASIEQPPDTMYLQMKRPLDMNSLENGPPTRKVIRIDAGTPDIQTSASTLQTDSGGGGCNKDSVLLNLLLRGEDRVYGYKVNNMIGKTFSQAMLPNLTRQDCEVNAPIQANPLLQGRELLHALEGIKMGAKIVRPHL
